# Supplementary material for: Identification of Differentially Expressed miRNAs in Porcine Adipose Tissues and Evaluation of Their Effects on Feed Efficiency
Source: Genes (Basel). 2022 Dec 19;13(12):2406. doi: 10.3390/genes13122406 (PMC9778086; doi:10.3390/genes13122406)

Figure S1. Length disribution of the miRNA in high-FE and low-FE pig adipose libraries.

This figure show the percentage distribution of the read lengths against the total counts in the miRNA reads of High-FE and Low-FE pig adipose libraries .

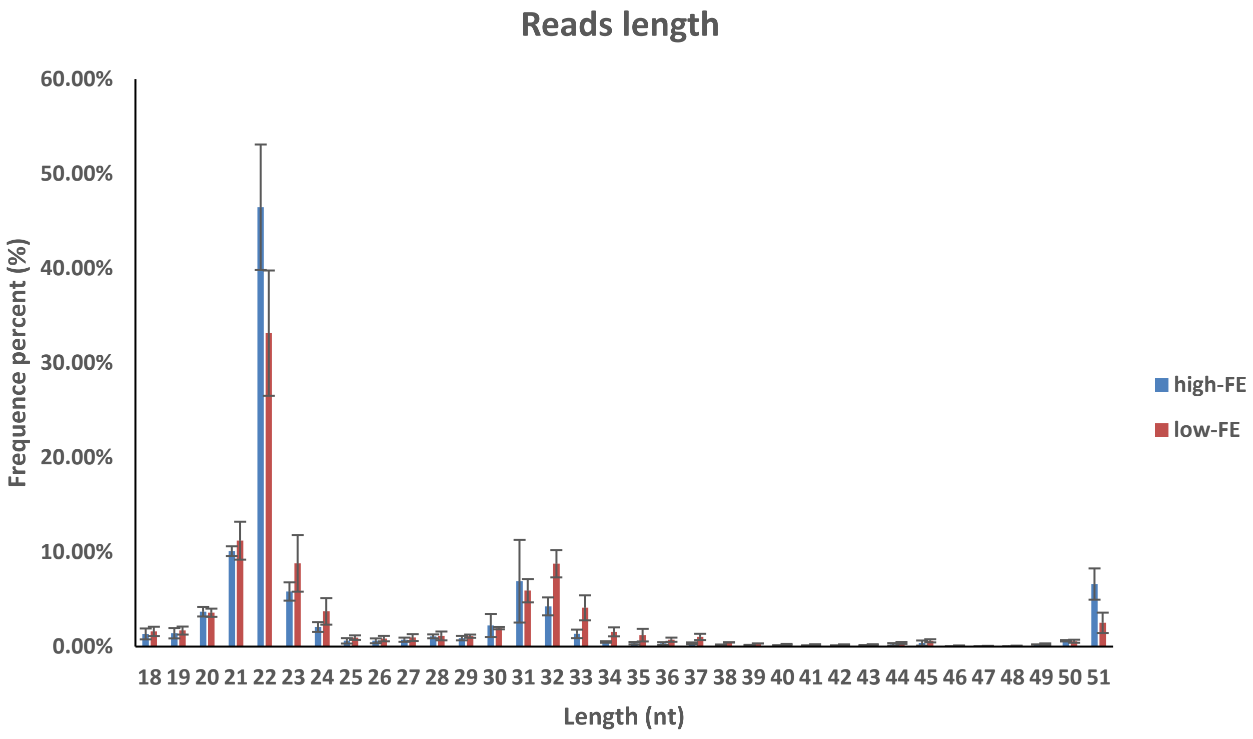

Supplement: Supplementary file 1 [file genes-13-02406-s001.zip › Figure S1.pdf]
